# Bacterial infection elicits the *Aedes aegypti* unfolded protein response

**DOI:** 10.1098/rsob.250207

**Published:** 2025-11-12

**Authors:** Dom Magistrado, Sarah M. Short

**Affiliations:** ^1^ Department of Entomology, The Ohio State University, Columbus, OH, USA

**Keywords:** unfolded protein response, stress response, mosquito, tolerance, homeostasis, infection

## Abstract

The unfolded protein response (UPR) is an ancient, highly conserved homeostatic cellular stress response pathway with diverse functions that include, but are not limited to, alleviating stress resulting from the presence of unfolded proteins in the endoplasmic reticulum of cells. Maintaining homeostasis and managing stress are critical to infection tolerance (i.e. host ability to mitigate infection-induced disease independently of strategies involving pathogen elimination). Stress responses such as the UPR are general mediators of tolerance, and the UPR may be activated during infections to promote host health. Understanding tolerance is an emerging priority in animal immunity, and there is unique motivation to understand how disease vectors tolerate infections because tolerance has implications for the efficiency of human pathogen transmission. However, stress responses are scarcely studied in arthropods, and the UPR has not been investigated in the context of a systemic mosquito infection. Herein, we characterize the trajectories of mortality and UPR transcript abundance in *Aedes aegypti* in response to infection with the opportunistic bacterial pathogen *Serratia marcescens*. We reveal that, with the exception of *atf6*, which displayed comparatively delayed activation, transcript levels of all UPR genes we measured harmoniously activate, peak, then diminish prior to the advent of appreciable infection-induced mortality.

**Figure:**
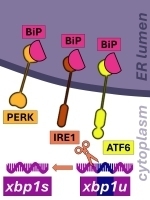

## 1. Introduction

Eukaryotic cells manage endoplasmic reticulum (ER) stress by activating a highly conserved collection of cellular signalling pathways known as the unfolded protein response (UPR). As a critical mechanism for maintaining cellular proteostasis, the UPR senses, responds to and alleviates ER stress resulting from the accumulation of unfolded or misfolded proteins in the ER lumen [1–3]. The UPR is comprised of three main branches, named for the three ER transmembrane proteins that govern distinct signalling networks: protein kinase R (PKR)-like kinase (PERK), activating transcription factor 6 (ATF6) and inositol-requiring enzyme-1 (IRE1), the last of which mediates the unconventional (i.e. independently of the spliceosome) splicing of *xbp1* mRNA [3,4]. At homeostasis, IRE1, PERK and ATF6 are bound to the ER resident chaperone protein BiP. Canonical activation of the UPR occurs when BiP is recruited away to bind misfolded proteins, and dissociation from BiP leaves IRE1, PERK and ATF6 unbound and therefore active [2,5–11]. However, more recent work substantiates an alternative BiP-independent mode of IRE1 and PERK activation involving the direct binding of misfolded proteins (in which misfolded proteins function as activating ligands) [2,12–20]. While the mode(s) of IRE1 and PERK activation are subject to debate and may be context-dependent, ATF6 is known only to be activated through dissociation from BiP.

The UPR functions to alleviate ER stress resulting from the accumulation of unfolded or misfolded protein by upregulating protein folding machinery and quality control measures, attenuating protein translation to reduce demands placed upon the ER, enhancing the self-repair ability of the ER and accelerating endoplasmic reticulum-associated protein degradation (ERAD)-mediated disposal of unfolded or misfolded proteins [1,2,21–23]. If the capacity of the UPR to alleviate stress is insufficient, the UPR initiates cellular apoptosis [24,25]. Canonically, the pathway functions as a feedback loop quality control system for restoring protein folding fidelity in cells through these channels. While the UPR is named for this critical and highly specific function, decades of research have revealed that the UPR is a pathway of paramount homeostatic importance that engages in cross-talk with other major biological pathways and is involved in a large suite of functions extending beyond addressing the accumulation of misfolded or unfolded proteins in cells. For example, recent work implicates the UPR as a master coordinator of cell homeostasis not only through the canonical protein quality control for which it is named, but also through regulating lipid metabolism to maintain lipid bilayer cell membranes through distinct signalling [26]. Therefore, the UPR is best understood as a core homeostatic pathway with critical functions that include, but are not limited to, alleviating stress resulting from the presence of unfolded or misfolded proteins in the ER.

The UPR has been implicated in a wide variety of physiological processes and helps organisms respond to a variety of endogenous and exogenous stressors. The UPR is indispensable for maintaining homeostasis during the routine stress of growth and development occurring early in life; in mice, medaka fish, *Xenopus laevis*, *Caenorhabditis elegans* and *Drosophila melanogaster*, UPR inactivation can lead to widespread developmental defects and embryonic mortality [27–32]. In addition to development, the UPR has been implicated in key functions related to metabolism, reproduction, senescence, non-infectious disease and immunity [33–42]. Furthermore, the UPR is involved in responses to various stressors, including toxins/pollutants (e.g. heavy metals, silica nanoparticles, pesticides and formaldehyde), pharmacological drugs (e.g. antibiotics and protease inhibitors), physical stressors (e.g. hypoxia, nutrient deprivation, thermal and pH stress) and infections [39–46].

Stress and damage resulting from infections can be caused by the invading microbe or by the host’s own immune system (i.e. immunopathology). While animal immunity has historically focused on the pathogen-killing aspect of the host’s response to infection (i.e. infection resistance), an equally important aspect of defence involves tactics for mitigating infection-induced damage and withstanding the physiological stress of infection that promote the health of the host (i.e. infection tolerance) [47,48]. As such, tolerance is inextricably linked to the maintenance of homeostasis during the stress of infection and is conceivably mediated by stress responses like the UPR [47]. Tolerance is an emerging priority in animal immunity; although it is currently unknown whether the molecular mechanisms governing tolerance are conserved, the general circuitry of tolerance is thought to be common across the animal kingdom [49]. Because homeostatic stress responses like the UPR are ancient, pervasive and have shaped successful interactions between organisms and stressful environments since the origin of the first cell [50], stress responses like the UPR are general mediators of stress tolerance during infections [47]. In the model organism *C. elegans*, XBP1 protects the host from immunopathology resulting from mounting an immune response against *Pseudomonas aeruginosa* [51,52], thereby promoting infection tolerance [47,49]. Further investigation of the UPR in infection and immunity contexts may implicate this pathway in tolerance circuits more broadly.

There is recent and unique motivation to understand tolerance in mosquitoes because tolerance predicates vector-borne disease transmission [53,54], and higher tolerance is associated with higher disease transmission and increased vectorial capacity in natural mosquito populations [55]. While foundational information about the mosquito UPR is sorely lacking, existing studies reveal functions of the pathway that are highly characteristic of tolerance: in one study, the *Ae. aegypti* UPR was activated upon ingestion of pore-forming toxins, and silencing of *ire1* or *xbp1* led to hypersensitivity to toxins and increased mortality [56]. Furthermore, the UPR is reportedly activated in response to blood feeding in mosquitoes [57], and we recently showed that blood feeding mitigates a decline in tolerance to bacterial infection in *Ae. aegypti* [58]. Taken together, these studies provide further support for the notion that the UPR could mediate tolerance in mosquitoes. To our knowledge, the UPR has not been investigated in the context of any *in vivo* mosquito infection. Herein, we identified mRNA homologues of all main branches of the UPR (*bip*, *ire1*, *perk*, *atf6* and both spliced and unspliced isoforms of *xbp1*) in *Ae. aegypti* and query the activity of the pathway during acute systemic infection with the cosmopolitan bacterium *Serratia marcescens*, a model opportunistic pathogen [59–63], across an infection time course. In addition, in an effort to establish a treatment with which the UPR may be activated in *Ae. aegypti* independently of infection, we explore the effect of oral and systemic exposure to dithiothreitol (DTT), a canonical UPR activator that was reported to activate the UPR in various insect systems [30,57,64], on UPR signalling in *Ae. aegypti*. Overall, this work elucidates the role of the UPR in *Ae. aegypti* in response to a systemic bacterial infection across time. The findings underscore the value of considering a host’s response to infection from a tolerance perspective and provide a foundation for future works to investigate the UPR as a tolerance mechanism.

## 2. Methods

### 2.1. Mosquitoes

*Ae. aegypti* Thai strain mosquitoes were maintained at 27°C and 80% relative humidity under a 14 h:10 h light:dark cycle. Eggs were hatched in RO (reverse osmosis) water placed in a vacuum chamber. Upon hatching, larvae were reared in trays containing RO water at a density of 200−300 larvae per tray and given one pinch of Tetramin fish flakes as well as cat food ad libitum until pupation and subsequent eclosion. Unless otherwise specified, adults were provided with a filter-sterilized 10% sucrose solution ad libitum through filter paper wick. In all experiments, mosquitoes were aged 3−6 days post-eclosion.

### 2.2. *Serratia marcescens* culture and infection treatment

Mosquitoes were infected with *Serratia marcescens* pPROBE-GFP. *Serratia marcescens* was isolated from *Anopheles gambiae* as described in [65] and transformed with a pPROBE-kanamycin plasmid containing a PnptII-GFP fusion construct [66] to obtain *Serratia marcescens* pPROBE-GFP (Dimopoulos Lab, unpublished data). Colonies of this strain of *S. marcescens* are tan in appearance when cultured with Luria broth (LB) but fluoresce green when exposed to light in the appropriate excitation range (we used 440−460 nm). Bacteria were grown in LB supplemented with kanamycin (50 µg ml^−1^) overnight at 30°C with shaking. Cultures were washed thrice in sterile 1× PBS, then pelleted and resuspended to OD600 = 1 ± 0.1 as measured with a BioSpectrometer (Eppendorf, Hamburg, Germany). At the time of infection, mosquitoes were anaesthetized on ice and injected with 69 nl of a 1:600 dilution of culture in sterile 1× PBS by piercing the soft tissue of the anepisternal cleft of the mesothorax using a Nanoject ii Auto-Nanoliter Injector fitted with a pulled glass needle. Control mosquitoes were injected with sterile 1× PBS. Fresh injection needles were prepared on each day that injections were performed by manually pulling a borosilicate glass capillary tube (Drummond, Broomall, PA, USA) over a flame to achieve a tip with an outer diameter no greater than 500 μm as measured using a stage micrometer. Following injections, mosquitoes were placed in paper cups and provided with a filter-sterilized 10% sucrose solution ad libitum. Mortality was monitored and mosquito tissues were collected at 0, 6, 12, 18, 24 and 48 h post-treatment. Samples consisted of *n* = 4–5 pooled whole mosquito bodies with heads removed. Samples were immediately homogenized in RLT (lysis) buffer from a Qiagen RNeasy Mini Kit (Qiagen no. 74104) and frozen at −80°C until RNA extraction. Six biological replicates were collected across two replicate batches. For replicates within a batch, infections were performed on the same day.

### 2.3. Injected dithiothreitol treatment

Mosquitoes were cold-anaesthetized and injected with DTT dissolved in filter-sterilized 1× PBS using a Nanoject ii Auto-Nanoliter Injector (Drummond, Broomall, PA, USA) set to deliver 69 nl. A serial dilution series was performed to obtain the following concentrations of DTT in filter-sterilized 1× PBS: 1 × 10^2^, 1 × 10^0^, 1 × 10^–2^, 1 × 10^–4^, 1 × 10^–6^ and 1 × 10^–8^ mg ml^−1^. DTT solutions were prepared immediately prior to use in experiments. A PBS-injected control group was used to account for effects of injection and an uninjected control group was used to account for the effects of cold anaesthesia and age on transcript abundance. Mosquito tissues were collected at 0, 6, 12, 18, 24, and 48 h post-treatment. Samples consisted of *n* = 4 pooled whole mosquito bodies with heads. Samples were immediately homogenized in RLT (lysis) buffer from a Qiagen RNeasy Mini Kit and frozen at −80°C until RNA extraction. Two biological replicates were collected.

### 2.4. Oral dithiothreitol treatment

For oral DTT experiments, mosquitoes were exposed to either: (i) a control treatment meal composed of filter-sterilized 10% sucrose + 5% (v/v) blue food dye (FD&C Blue No. 1, Ward’s Science no. 470057-798) or (ii) a DTT treatment meal composed of filter-sterilized 10% sucrose + 5% (v/v) blue food dye + 0.025% (w/v) DTT. Control or DTT treatment meals were prepared immediately prior to use in experiments and were delivered to mosquitoes by soaking cotton balls with the solutions, then placing the cotton balls on top of mesh netting of the mosquito cages. Exposure to meals lasted 2.5 h before removal and replacement with standard filter-sterilized 10% sucrose. Ingestion of meals was confirmed by the presence of blue abdomens upon visual inspection in all experimental mosquitoes (electronic supplementary material, figure S1). The 0.025% concentration of DTT in the meal was selected by performing a dose–response experiment exposing mosquitoes to a range of sugar meal DTT concentrations to determine dose thresholds for mortality and/or feeding propensity. The selected concentration of 0.025% was the highest dose which did not cause mortality and was readily ingested by mosquitoes. Mosquito tissues were collected at 6, 12, 18, 24 and 48 h post-treatment. Mosquitoes were cold-anaesthetized before dissections. The midguts and crops were dissected and combined. Heads were removed from the remaining carcasses and discarded. Pooled samples consisted of *n* = 3–5 individual headless carcasses or *n* = 3–5 individual combined midguts and crops (in which *n* = 1 is one combined midgut and crop from an individual mosquito) per sample. Samples were immediately homogenized in RLT (lysis) buffer from a Qiagen RNeasy Mini Kit and frozen at −80°C until RNA extraction. Three biological replicates were collected.

### 2.5. RNA extraction and RT-qPCR

RNA was extracted from tissue samples using a Qiagen RNeasy Mini Kit (Qiagen no. 74104) per the manufacturer’s instructions. DNA contamination was removed from RNA samples using a TURBO™ DNase kit (Invitrogen™ no. AM2238) per the manufacturer’s instructions for Rigorous DNAse treatment. Nucleic acid concentrations in the samples were measured with a NanoDrop One spectrophotometer and standardized before cDNA synthesis. cDNA was synthesized using M-MLV Reverse Transcriptase (Promega no. M1701) and oligo dT primers. Each RT-qPCR assay reaction used 400 ng of RNA. Samples were diluted 1:10 following cDNA synthesis.

qPCR assay reactions targeted housekeeping gene (*s7*) and UPR genes: *bip*, *ire1*, *perk*, *atf6*, *xbp1u* (the unspliced isoform of *xbp1*) and *xbp1s* (the spliced isoform of *xbp1*) (electronic supplementary material, table S1). Reactions were performed on a Bio-Rad CFX96 Touch Deep Well Real-Time PCR Detection System in a volume of 10 μl containing 0.5 μl of each 10 μM primer, 4 μl of cDNA template diluted 1:10, and 5 μl of SYBR^®^ Green PCR Master Mix (Life Technologies no. 4309155). Electronic supplementary material, table S1, includes information on genes and primers, including VectorBase accession numbers, primer sequences, product sizes, optimized annealing temperatures (*T*_A_), baseline threshold used for each qPCR assay and qPCR assay efficiency. Cycling parameters were: 2 min at 95°C hot start + (15 s at 95°C+1 min at *T*_A_) × 40 cycles, followed by a 0.5°C resolution melt curve analysis. The primers targeting *s7* were obtained from Sim *et al*. [67]. Primers for UPR genes were designed de novo using Primer3 and NCBI Primer BLAST. We performed melt curve analysis and agarose gel electrophoresis on representative qPCR products for all genes to validate the presence of a single product of the expected size. Furthermore, qPCR products for *bip*, *ire1*, *perk*, *xbp1s* and *xbp1u* were subjected to Sanger sequencing for further validation. For each qPCR assay designed, reaction efficiencies were calculated using standard curves collected from at least three technical replicates. Standard curve dilution series were generated over a sufficiently wide range such that sample *Cq* values fell within the range of *Cq* values in the standard curves, with an additional 1 *Cq* margin for buffer. Melt curve analysis was performed on all sample reactions and confirmed the presence of a single peak. Duplicate technical replicates were performed per sample per gene of interest with an inclusion criterion of 0.5 *Cq* distance from the average.

### 2.6. Statistical analysis

All statistical analyses were performed using R statistical software (v4.4.1; R Core team 2024) and RStudio (v4.2.764; Posit team 2024). All data can be found in electronic supplementary material, file S1. All R code can be found in electronic supplementary material file S2.

We used the *X*_0_ method [68] to compute qPCR relative transcript abundance values. Through this method, *Cq* values are subjected to efficiency-specific (electronic supplementary material, table S1, *E*_Amp_) exponential decay transformations resulting in qPCR assay efficiency-corrected, linearly related *X*_0_ values representative of the amount of starting transcript in samples per equation (2.1). Gene of interest (GOI) *X*_0_ values were then expressed relative to housekeeping gene *s7 X*_0_ values to obtain Δ*X*_0_ values, which were used for all analyses per equation (2.2). We also calculated *xbp1s*_proportion_, a value which represents the proportion of *xbp1s* out of the total *xbp1* mRNA (i.e. *xbp1s* and *xbp1u*), using equation (2.3). This is a suitable method for inter-gene proportion calculations, as *X*_0_ values (unlike *Cq* values) are linearly related and are suitably representative of the starting amount of transcript in a qPCR assay reaction.


(2.1) $$X_{0}=(1+E_{Amp}{)}^{-Cq},$$

(2.2) $$∆X_{0,GOI}=X_{0,GOI}/X_{0,s7},$$

(2.3) $${xbp1s}_{\text{proportion}}=X_{0,xbp1s}/(X_{0,xbp1s}+X_{0,xbp1u}).$$

We used *a priori* contrasts to inquire a statistically significant effect of each treatment (*Serratia marcescens* infection treatment, injected DTT treatment and oral DTT treatment) at each timepoint. *A priori* contrasts are appropriate in this setting because we originally hypothesized that transcript abundance may vary between the treatment and control groups at one or more timepoints [69]. We stratified the dataset by gene of interest and built nested ANOVAs in which treatment was nested within time for each gene, then performed contrasts on Δ*X*_0,GOI_ values comparing all levels of non-control treatment variables to the control level of the treatment variable using the R package emmeans [70] with the default *p*-value adjustments for multiple testing. A natural log transformation was applied to only the Δ*X*_0,*bip*_ vaues from the infection treatment dataset, as the transformation improved the normality (assessed qualitatively by visual inspection) of the data distribution. We also confirmed the absence of any effect of treatment on the housekeeping gene *s7*, a commonly used reference gene in *Ae. aegypti* gene expression experiments [71], using contrasts in a manner similar to that used for the UPR genes (i.e. we built nested ANOVAs in which treatment was nested within time, then performed contrasts on *X*_0,_*_s7_* values as described in the preceding paragraph). We detected no effect of treatment at any timepoints (electronic supplementary material, file S2).

We performed survival analysis to query the effect of bacterial infection on mosquito mortality across the infection time course. The Kaplan–Meier method was used to estimate the probability of mosquito survival post-infection. The log-rank test was used to determine statistically significant differences between groups.

### 2.7. Relative expression profiles

For genes *bip*, *ire1*, *perk*, *atf6*, *xbp1s* and *xbp1u*, transcript abundance values for *S. marcescens*-infected samples were expressed relative to PBS injury control samples by dividing within-batch averages for *S. marcescens*-infected samples by within-batch averages for PBS injury control samples at each time point to obtain relative values for each batch, then averaging relative batch values at each time point. For *xbp1s* (as proportion), because these values are proportions, we subtracted within-batch averages for PBS injury control samples from within-batch averages for *S. marcescens*-infected samples, then averaged relative batch values at each time point. Electronic supplementary material, file S2, contains all calculations and R code used to create the relative expression profiles.

## 3. Results

### 3.1. Systemic infection with *Serratia marcescens* causes acute mortality and activates the *Aedes aegypti* unfolded protein response

**Figure 1.**
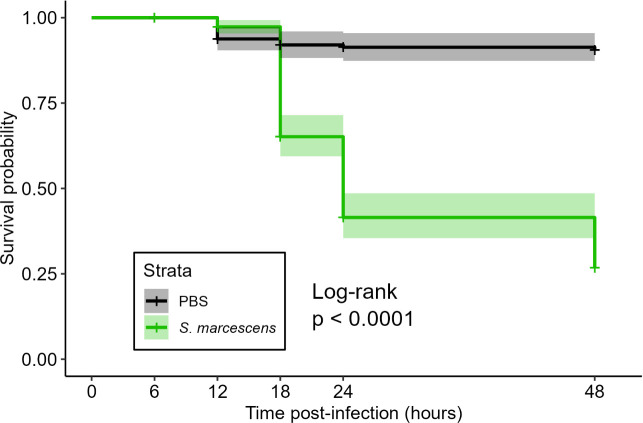
Mosquito survival is significantly reduced by infection with *Serratia marcescens*. The Kaplan–Meier method is used to estimate probability of survival post-infection. Shaded areas surrounding curves represent 95% confidence intervals. The log-rank test revealed a statistically significant reduction in the probability of survival of *S. marcescens*-infected mosquitoes compared with PBS injury control mosquitoes.

**Figure 2.**
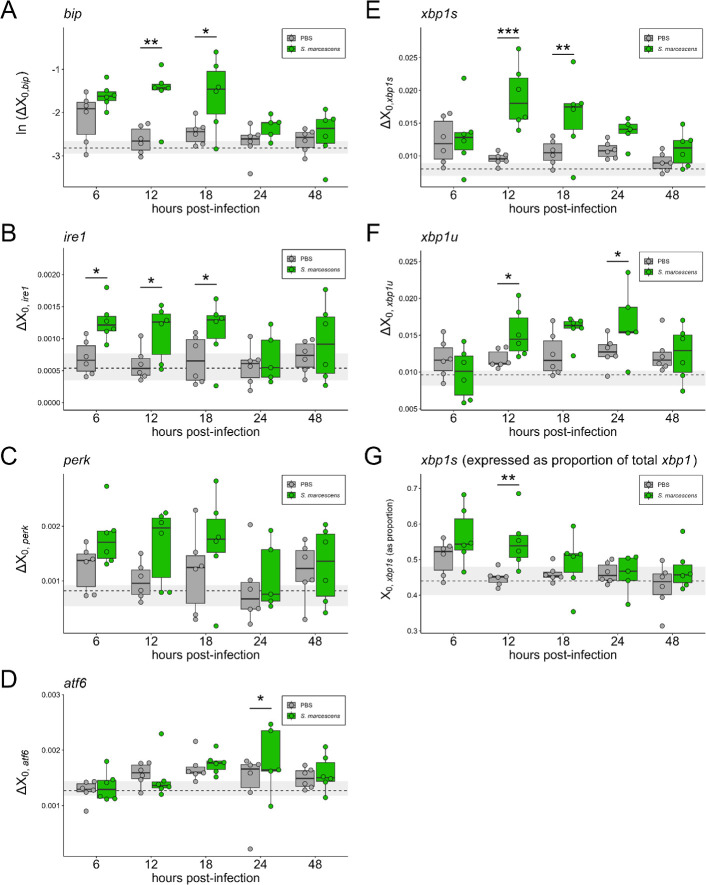
Transcript abundance of UPR genes across time in *S. marcescens*-infected and PBS injury control groups. In (A–F), *Y*-axis values are Δ*X*_0_ values representing the amount of (A) *bip* (natural log-transformed), (B) *ire1*, (C) *perk*, (D) *atf6*, (E) *xbp1s*, or (F) *xbp1u* transcript in samples, standardized to housekeeping gene *s7* (i.e. Δ*X*_0,GOI_ = *X*_0,GOI_/*X*_0,_*_s7_*). In (G), *Y*-axis values represent the proportion of *xbp1s* (spliced isoform) transcript in samples relative to total *xbp1* transcript per equation (2.3). Individual points represent pooled samples of *n* = 4–5 mosquito whole bodies with heads removed. Boxplots displaying the median and interquartile range were constructed from plotted points. The dotted horizontal line and grey shading display the median and interquartile range of *n* = 11–12 pooled samples of *n* = 4–5 uninjured individuals sampled at 0 h post-infection and serve as a visual indicator of pre-treatment, baseline transcript abundance. Significance codes represent the outcome of pairwise comparisons of PBS versus *S. marcescens*-injected groups at each timepoint. *p < 0.05, **p < 0.01, ***p < 0.001.

We investigated the effect of systemic infection with *S. marcescens* on the UPR in female Thai strain *Ae. aegypti* mosquitoes across time. Infection with *S. marcescens* led to rapid mortality within the first 24 hours post-infection (hpi), with most mortality occurring between 12 and 24 hpi (figure 1). Infection with *S. marcescens* induced significant or marginally significant increases in transcript abundance for UPR genes *bip* (figure 2, table 1: *p_bip_*_, 12hpi_ = 2.00 × 10^−4^, *p_bip_*_, 18hpi_ = 0.003), *ire1* (figure 2, table 1: *p_ire1_*_, 6hpi_ = 0.013, *p_ire1_*_, 12hpi_ = 0.028, *p_ire1_*_, 18hpi_ = 0.043) and *atf6* (figure 2, table 1: *p_atf6_*_, 24hpi_ = 0.050), as well as the spliced and unspliced isoforms of *xbp1* (*xbp1s* and *xbp1u*, respectively) (figure 2, table 1: *p_xbp1s_*_, 12hpi_ = 1.52 × 10^−5^, *p_xbp1s_*_, 18hpi_ = 0.006, *p_xbp1u_*_, 12hpi_ = 0.041, *p_xbp1u_*_, 18hpi_ = 0.053, *p_xbp1u_*_, 24hpi_ = 0.036). Infection with *S. marcescens* also induced significant increases in the relative proportion of *xbp1* transcripts that were *xbp1s* (figure 2, table 1: *p_xbp1s_*__proportion, 12hpi_ = 0.004). *ire1* was the only gene to display a significant increase in response to infection at 6 hpi. 12 hpi showed the most significant pairwise differences, with *bip*, *ire1*, *xbp1s*, *xbp1u* and *xbp1s*_proportion_ all showing significant increases in response to infection. *perk* displayed a marginally significant increase only at 12 hpi (table 1: *p_perk_*_, 12hpi_ = 0.076) while *atf6* displayed a marginally significant increase only at 24 hpi (table 1: p*_atf6_*_, 24hpi_ = 0.050). No genes showed any significant differences at 48 hpi.

**Table 1.** The effect of *S. marcescens* infection treatment on *bip*, *ire1*, *perk*, *atf6*, *xbp1s*, *xbp1u* and *xbp1s_proportion_*: contrasts comparing the *S. marcescens* treatment to the PBS control, nested within time.

time (hpi)	contrast	estimate	s.e.	*T*-ratio	*p*‐value	
*bip*						
6	PBS versus *S. marcescens*	−0.51	2.77 × 10^−^¹	−1.849	0.071	
12	PBS versus *S. marcescens*	−1.10	2.77 × 10^−1^	−3.969	**2.0 × 10^−4^**	**
18	PBS versus *S. marcescens*	−0.88	2.77 × 10^−1^	−3.165	**0.003**	*
24	PBS versus *S. marcescens*	−0.36	2.90 × 10^−1^	−1.223	0.227	
48	PBS versus *S. marcescens*	−0.13	2.77 × 10^−1^	−0.466	0.643	
*ire1*						
6	PBS versus *S. marcescens*	−5.7 × 10^−4^	2.19 × 10^−4^	−2.588	**0.013**	*
12	PBS versus *S. marcescens*	−5.0 × 10^−4^	2.19 × 10^−4^	−2.259	**0.028**	*
18	PBS versus *S. marcescens*	−4.6 × 10^−4^	2.19 × 10^−4^	−2.076	**0.043**	*
24	PBS versus *S. marcescens*	−1.2 × 10^−4^	2.30 × 10^−4^	−0.527	0.601	
48	PBS versus *S. marcescens*	−2.3 × 10^−4^	2.19 × 10^−4^	−1.035	0.306	
*perk*						
6	PBS versus *S. marcescens*	−5.5 × 10^−4^	3.62 × 10^−4^	−1.509	0.138	
12	PBS versus *S. marcescens*	−6.6 × 10^−4^	3.62 × 10^−4^	−1.815	0.076	
18	PBS versus *S. marcescens*	−5.4 × 10^−4^	3.62 × 10^−4^	−1.491	0.142	
24	PBS versus *S. marcescens*	−2.4 × 10^−4^	3.80 × 10^−4^	−0.626	0.534	
48	PBS versus *S. marcescens*	−1.0 × 10^−4^	3.62 × 10^−4^	−0.282	0.779	
*atf6*						
6	PBS versus *S. marcescens*	−8.4 × 10^−5^	2.03 × 10^−4^	−0.414	0.681	
12	PBS versus *S. marcescens*	−7.3 × 10^−5^	2.03 × 10^−4^	0.358	0.722	
18	PBS versus *S. marcescens*	−7.7 × 10^−5^	2.03 × 10^−4^	−0.380	0.706	
24	PBS versus *S. marcescens*	−4.3 × 10^−4^	2.03 × 10^−4^	−2.008	**0.050**	*
48	PBS versus *S. marcescens*	−8.9 × 10^−5^	2.03 × 10^−4^	−0.437	0.664	
*xbp1s*						
6	PBS versus *S. marcescens*	−7.4 × 10^−4^	1.98 × 10^−4^	−0.372	0.711	
12	PBS versus *S. marcescens*	−9.5 × 10^−3^	1.98 × 10^−4^	−4.803	**1.52 × 10^−5^**	***
18	PBS versus *S. marcescens*	−5.7 × 10^−5^	1.98 × 10^−4^	−2.867	**0.006**	**
24	PBS versus *S. marcescens*	−2.8 × 10^−3^	2.07 × 10^−4^	−1.343	0.186	
48	PBS versus *S. marcescens*	−2.8 × 10^−3^	1.98 × 10^−4^	−1.006	0.319	
*xbp1u*						
6	PBS versus *S. marcescens*	−2.0 × 10^−3^	1.72 × 10^−3^	1.154	0.254	
12	PBS versus *S. marcescens*	−3.6 × 10^−3^	1.72 × 10^−3^	−2.095	**0.041**	*
18	PBS versus *S. marcescens*	−3.4 × 10^−3^	1.72 × 10^−3^	−1.982	0.053	
24	PBS versus *S. marcescens*	−3.9 × 10^−3^	1.80 × 10^−3^	−2.151	**0.036**	*
48	PBS versus *S. marcescens*	−1.2 × 10^−3^	1.72 × 10^−3^	−0.071	0.944	
*xbp1s_proportion_*						
6	PBS versus *S. marcescens*	−0.060	0.034	−1.779	0.081	
12	PBS versus *S. marcescens*	−0.102	0.034	−3.039	**0.004**	**
18	PBS versus *S. marcescens*	−0.029	0.034	−0.880	0.383	
24	PBS versus *S. marcescens*	0.003	0.035	0.097	0.923	
48	PBS versus *S. marcescens*	−0.048	0.034	−1.445	0.155	

**Figure 3.**
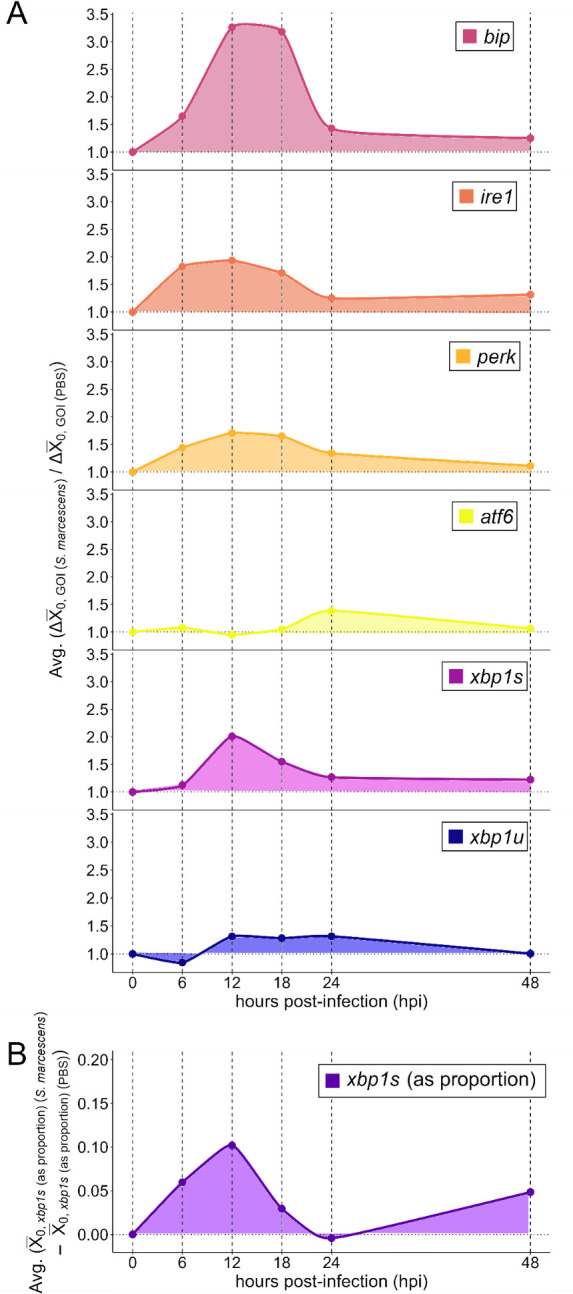
Relative expression profiles displaying *S. marcescens*-induced activation of UPR genes expressed relative to PBS injury controls across time. (A) Average *bip*, *ire1*, *perk*, *atf6*, *xbp1s*, and *xbp1u* transcript abundance values and (B) average *xbp1s* (as proportion) values for *S. marcescens*-infected samples were expressed relative to average PBS injury control samples at each timepoint within batch via subtraction (for proportion values) or via division (for all other values), then relative values for each batch were averaged and plotted. *X*_0,_*_xbp1s_*_(as proportion)_ values represent the proportion of *xbp1s* (spliced isoform) transcript in samples relative to total *xbp1* transcript per equation (2.3).

To further understand the temporal effects of infection on UPR induction across all tested genes, we expressed average Δ*X*_0,GOI_ values (averages within each batch) relative to those of the injury control and plotted smoothed curves across time to generate a stacked temporal transcript abundance profile (figure 3). This analysis reveals temporal parallels in the responses of *bip*, *ire1*, *perk*, and *xbp1s*. The activation profile of *atf6* was markedly different from that of the other genes, displaying a distinct peak at 24 hpi that is relatively small compared with the other genes but consistent between batches (electronic supplementary material, figure S2). Taken altogether, the data reveal an acute infection-induced increase in *Ae. aegypti* UPR transcript production that begins prior to appreciable mortality.

### 3.2. Treatment with canonical chemical unfolded protein response inducer dithiothreitol does not systemically activate unfolded protein response genes *ire1* and/or *bip* in *Aedes aegypti*

We delivered canonical pharmacological UPR inducer DTT to mosquitoes using two methods: (i) microinjection and (ii) orally through a DTT-supplemented sugar meal.

**Figure 4.**
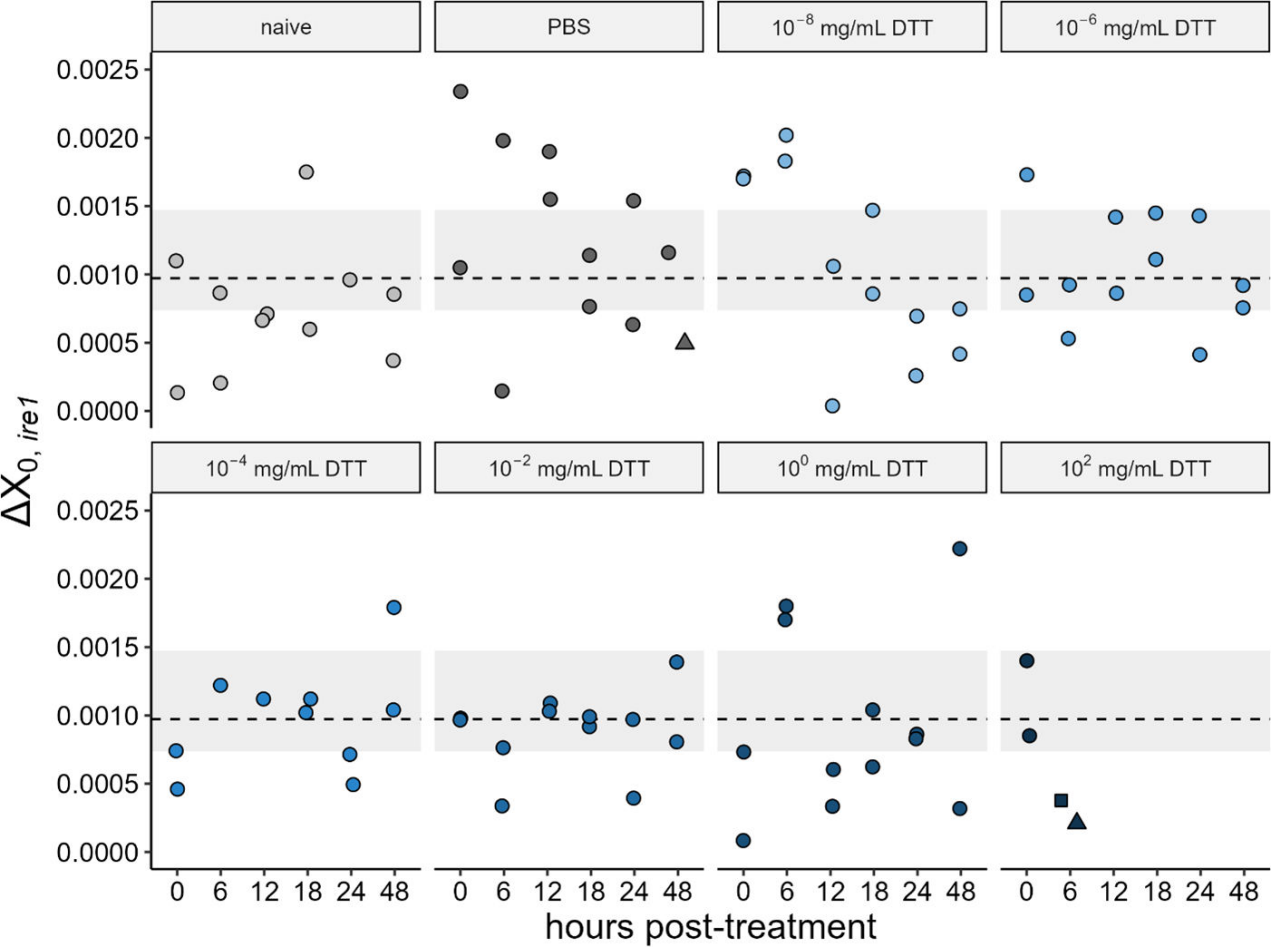
Transcript abundance of *ire1* across time in naive, PBS-injected, and DTT-injected mosquitoes. *Y*-axis values are Δ*X*_0_ values representing the amount of *ire1* transcript present in samples, standardized to housekeeping gene *s7* (i.e. Δ*X*_0,GOI_ = *X*_0,GOI_/*X*_0,*s7*_). Individual points represent pooled samples of *n* = 3–5 mosquito whole bodies with heads and legs removed, with the exception of two triangle points (representing pools of *n* = 2) and one square point (representing *n* = 1 individual). The dotted horizontal line and grey shading display the median and interquartile range of the *n* = 16 pooled samples of *n* = 3–5 naive individuals sampled at 0 h post-DTT injection and serve as a visual indicator of pre-treatment, baseline transcript abundance. Data were collected across two biological replicates.

Using microinjection, we exposed mosquitoes to DTT doses ranging from 1 × 10^–8^ to 1 × 10^2^ mg ml^–1^ DTT delivered to the hemocoel through injection to query a systemic UPR dose response to injected DTT. Mosquitoes injected with the highest dose were nearly all dead by 6 h (21 of 22 dead in replicate 1, 17 of 19 dead in replicate 2). Individuals still alive at 6 h were sampled and subjected to RT-qPCR alongside all other samples. No other treatment or control groups displayed considerable mortality at any timepoint. Contrasts between each treatment group and the injury control group at each timepoint revealed no effect of DTT injection on *ire1* transcript abundance at any timepoint (figure 4; electronic supplementary material, table S2).

**Figure 5.**
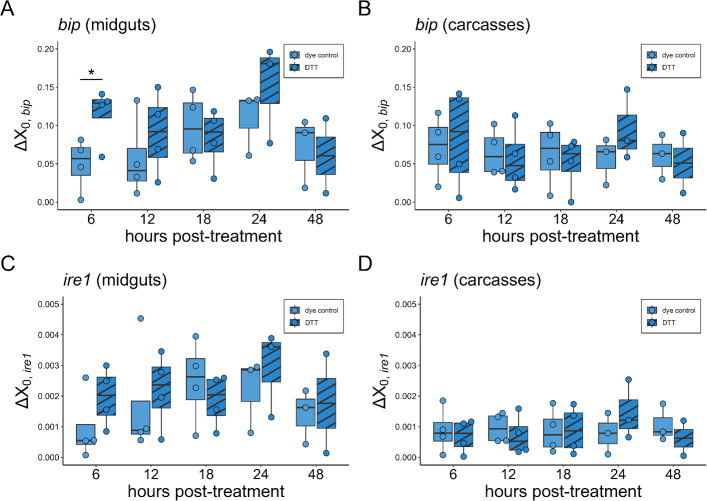
Effect of oral DTT treatments on transcript abundance of *bip* (A,B) and *ire1* (C,D) in mosquito midguts (A,C) or carcasses (B,D) across time. *Y*-axis values are Δ*X*_0_ values representing the amount of GOI transcript present in samples, standardized to housekeeping gene *s7* (i.e. Δ*X*_0,GOI_ = *X*_0,GOI_/*X*_0*,*_*_s7_*). Individual points represent pooled samples of *n* = 4–5 mosquito midguts or carcasses with heads removed. Significance codes represent the outcome of pairwise comparisons of oral DTT treatment at each timepoint. *p < 0.05.

Using oral exposure through sugar meals, we exposed mosquitoes to 0.025% DTT. Contrasts indicated that oral DTT exposure increased abundance of *bip* in the midgut but not the carcass at 6 h post-feeding (figure 5, table 2: *p_bip_*_, 6hpi_ = 0.048) but not at any other timepoints. Oral DTT exposure had no effect on abundance of *ire1* in either tissue at any timepoint (figure 5, table 2).

**Table 2.** The effect of oral DTT treatment on *ire1* and *bip* transcript abundance: contrasts comparing the DTT treatment to the dye control, nested within tissue and time.

time	tissue	contrast	estimate	s.e.	*T*-ratio	*p*‐value	
*bip*							
6	midgut	DTT versus dye control	−6.5 × 10^−2^	3.2 × 10^−2^	−2.03	**0.048**	*
6	carcass	DTT versus dye control	−1.1 × 10^−2^	3.2 × 10^−2^	−0.34	0.732	
12	midgut	DTT versus dye control	−3.3 × 10^−2^	3.2 × 10^−2^	−1.04	0.304	
12	carcass	DTT versus dye control	8.6 × 10^−3^	3.2 × 10^−2^	0.27	0.789	
18	midgut	DTT versus dye control	1.5 × 10^−2^	3.2 × 10^−2^	0.46	0.649	
18	carcass	DTT versus dye control	1.2 × 10^−2^	3.2 × 10^−2^	0.36	0.719	
24	midgut	DTT versus dye control	−4.2 × 10^−2^	3.7 × 10^−2^	−1.14	0.261	
24	carcass	DTT versus dye control	−3.9 × 10^−2^	3.7 × 10^−2^	−1.05	0.299	
48	midgut	DTT versus dye control	1.1 × 10^−2^	4.1 × 10^−2^	0.26	0.797	
48	carcass	DTT versus dye control	9.3 × 10^−3^	4.1 × 10^−2^	0.23	0.822	
*ire1*							
6	midgut	DTT versus dye control	−1.0 × 10^−3^	7.4 × 10^−4^	−1.38	0.174	
6	carcass	DTT versus dye control	1.9 × 10^−4^	7.4 × 10^−4^	0.25	0.800	
12	midgut	DTT versus dye control	−4.8 × 10^−4^	7.4 × 10^−4^	−0.64	0.523	
12	carcass	DTT versus dye control	2.6 × 10^−4^	7.4 × 10^−4^	0.34	0.732	
18	midgut	DTT versus dye control	6.1 × 10^−4^	7.4 × 10^−2^	0.82	0.417	
18	carcass	DTT versus dye control	−4.1 × 10^−5^	7.4 × 10^−4^	−0.05	0.956	
24	midgut	DTT versus dye control	−7.3 × 10^−4^	8.6 × 10^−4^	−0.85	0.398	
24	carcass	DTT versus dye control	−6.9 × 10^−4^	8.6 × 10^−4^	−0.80	0.425	
48	midgut	DTT versus dye control	−3.5 × 10^−4^	9.6 × 10^−4^	−0.36	0.718	
48	carcass	DTT versus dye control	4.4 × 10^−4^	9.6 × 10^−4^	0.45	0.652	

## 4. Discussion

### 4.1. *Aedes aegypti* infected with *Serratia marcescens* display temporally dynamic unfolded protein response activation

Our data indicate that systemic infection with the opportunistically pathogenic bacterium *S. marcescens* transiently activates the *Ae. aegypti* UPR. Historically, the UPR has been well studied in the context of infections with viruses and obligate intracellular parasites and pathogens. Unlike *S. marcescens* which can thrive in the environment and replicate independently of a host, these pathogens usually lack endogenous protein processing machinery and therefore must hijack that of the host cell for replication [45,72–75]. By this virtue, activation of the host’s UPR by viruses and obligate intracellular parasites and pathogens is intuitive, as reliance on the host’s cells for protein processing imposes ER stress on the host due to increased demands. In contrast, the mechanism behind UPR induction by the opportunistic pathogen *S. marcescens* is less clear, although there are a variety of promising hypotheses.

Environmental *S. marcescens* is typically red in appearance due to the production of the secondary metabolite pigment prodigiosin, which has been shown to induce ER stress and activate the UPR in yeast and human cells [76–78]. The *S. marcescens* strain used in this study is tan in appearance when cultured on LB, suggesting that it lacks prodigiosin. Therefore, UPR induction observed in our results may be prodigiosin-independent. While prodigiosin has received significant attention due to potential for beneficial applications, clinically significant *S. marcescens* isolates causing nosocomial infections are overwhelmingly non-pigmented [79–81], and additional studies have revealed that prodigiosin is not an essential virulence factor for *S. marcescens* [63].

There are reports of opportunistic bacterial pathogens activating the UPR in their hosts. *Pseudomonas aeruginosa* produces virulence factors that directly activate multiple host stress responses in human epithelial cells, including the UPR [82]. Bacteria-derived pore-forming toxins (PFTs) activate the UPR in *Ae. aegypti* [56,83], *C. elegans* [84], *Manduca sexta* [83] and in mammalian cells [84], wherein UPR activity had a protective effect for the host. While the mechanism of *S. marcescens*-induced mosquito mortality is not known, *S. marcescens* produces PFTs [85–88] that may contribute to the mortality observed in our mosquitoes. Possibly, UPR activation in response to PFTs or other virulence factors serves to protect mosquitoes from *S. marcescens*.

Some bacteria hijack the host UPR for their own purposes, similar to viruses and other intracellular parasites. Studies on *Streptococcus pyogenes* provide evidence that toxins produced by these microbes activate the UPR in hosts, leading to the ATF6-mediated release of the nutrient asparagine that is critical for bacterial proliferation [89]. While the mechanism by which *S. marcescens* survives and proliferates within the mosquito host has not been reported, *S. marcescens* is internalized by various mammalian phagocytic and non-phagocytic cells *in vitro*, as well as in *D. melanogaster* midgut epithelial cells *in vivo* [90–95], and can survive and replicate in vacuoles within the cell. Interestingly, other bacterial pathogens that internalize and replicate in host cells are known to associate with the ER of the cells and coopt the organelle to promote bacterial survival and proliferation [96]. While this has not been demonstrated in *S. marcescens*, *S. marcescens* can manipulate host cell machinery in other settings. For example, the use of a distinct Ca^2+^ dependent strategy for reshaping the cytoskeleton permits nonlytic egress from mammalian host cells [91], an elegant tactic that may be beneficial to the bacteria by virtue of limiting infection-induced disease in the host (i.e. promoting tolerance). It is possible that *S. marcescens* internalizes into mosquito host cells and subverts the ER in a fashion similar to that seen in other host interactions, and the UPR activity we observed occurs in response to ER stress.

Both infection-induced mortality and infection-induced UPR activation were transient, as UPR transcript abundance levels returned to baseline by 48 h post-infection and mortality decelerated after 24 h post-infection. Furthermore, while we did not measure bacterial load in this experiment, previous data from our research group [59,97] show that mosquitoes infected with *S. marcescens* by microinjection do not clear their infections on a timeline concomitant with observed mortality, rather, many mosquitoes continue to harbour *S. marcescens* several days following infection. This suggests that following the acute mortality phase of infection, *S. marcescens* persists in a stage of chronic infection that seemingly no longer causes mortality or induces UPR activity. Taken together, these data suggest that the presence of *S. marcescens* in the body alone is unlikely to explain the UPR induction we observe. Plausibly, changes in bacterial load across time may correspond with the mRNA expression of one or more UPR genes. Alternatively, UPR induction may not be coupled with either mortality or bacterial load and rather, UPR induction may be inherently transient in this infection system, possibly due to niche switching [98] by *S. marcescens*. Additional work elucidating the mosquito host response and *S. marcescens*’ mechanism of survival and proliferation in *Ae. aegypti* would be required to fully understand the UPR activation findings reported herein.

### 4.2. Infection-induced activation of *ire1* and *perk* is distinct from that of *atf6*

In our data, we observed an increase in *atf6* transcripts concomitant with a decrease in *bip* transcripts at 24 h post-infection. In contrast, *ire1* and *perk* transcripts increased concomitantly with *bip*. The similarity of the mRNA expression profiles of *bip*, *ire1* and *perk*, combined with marked *atf6* incongruency, is interesting and invokes consideration of how each of the three main transmembrane signalling proteins are activated. Two classes of modes of activation have been reported in other systems: (i) a BiP-dependent mode class, in which IRE1, PERK and ATF6 are necessarily activated by dissociation from BiP [2,5–11], and (ii) a BiP-independent mode class, in which IRE1 and PERK can be activated by direct binding to misfolded proteins [2,12–20]. In all systems studied to date, ATF6 is activated only by the BiP-dependent mode. Importantly, the mechanism of UPR activation has not been explored in any insect system and thus remains unknown. In the current study, we measured transcripts and not protein abundance or activity. However, the unique nature of the transcriptional profile of *atf6* compared with *ire1*/*perk* motivates further investigation into whether BiP-dependency of ATF6 protein abundance and/or activity is different from that of IRE1 and/or PERK in mosquitoes.

The ATF6 branch of the UPR has recently been characterized as playing an important role in remodelling cell physiology and promoting recovery following acute physiological and pathological injury via the ‘adaptive UPR’ [99]. We observed *atf6* activation in parallel with deceleration of mosquito mortality at 24 h post-infection, so it is possible that ATF6 mediates tolerance to infection by coordinating the repair of *S. marcescens*-induced damages. Future experiments will determine the functional role of this under-investigated branch of the UPR in arthropods and other taxa in infection and disease contexts.

### 4.3. Treatment with dithiothreitol did not strongly activate *bip* and *ire1*

The results of the oral DTT exposure experiment showed that DTT ingestion induced a small but statistically significant increase (*p* = 0.048) in the abundance of *bip* in the midgut tissue at 6 h post-treatment but at no other times. We observed no significant increase in the abundance of *ire1* in the midgut at any timepoint. Oral DTT treatment had no effect on *bip* or *ire1* transcript abundance in carcasses at any timepoint post-treatment. DTT is a widely used reagent for stimulating the UPR, primarily in cell culture contexts, but also *in vivo* by feeding and by direct injection [57,64,100], and it can induce a dose-dependent effect on UPR activity [101]. Feeding honey bees 10 mM DTT for 8 h increases abundance of *bip*, total *xbp1* and *xbp1s* in the digestive tract [64], and incubating *D. melanogaster* tissues or S2 cells in media supplemented with 5 mM DTT (tissues) or 1 mM DTT (S2 cells) increases *xbp1* splicing [102]. We orally exposed mosquitoes to DTT or control meals for only 2.5 h. However, when mosquitoes imbibe nectar or other sugar meals, the solution is stored in a region of the digestive tract that generally does not contribute to nutrient absorption known as the crop, then directed to the midgut incrementally as needed [103–105]. Consistent with this, we observed during dissections that the mosquito midguts and/or crops were visibly blue throughout 24 h post-treatment (data not shown), suggesting that digestive tissues were exposed to DTT for much longer than the initial 2.5 h feeding window. Moreover, we exposed mosquitoes to a 0.025% DTT solution, equivalent to approximately 160 mM DTT. This is a relatively high dose of DTT compared with that used in previous studies in other systems [64,102]. Therefore, we find it very surprising that we did not observe increased transcript abundance of the UPR genes we tested.

Similarly, we found that a range of DTT doses delivered directly to the hemocoel of mosquitoes had no effect on the abundance of *ire1*. These data contrast with those reported by Weng & Shiao [57], who showed that DTT injection led to temporally dynamic increases in *bip*, *ire1* and *xbp1*, with particularly strong effects of DTT at 24 h on *ire1*, which displayed more than 100-fold increases relative to the PBS injury control group. The technical and/or biological explanations for these contrasting results are unclear. We welcome further investigation by other researchers to better elucidate this phenomenon.

## Data Availability

All data, code, and supporting materials can be accessed in the electronic supplementary material [106].
